# Enhancing antibacterial property of porous titanium surfaces with silver nanoparticles coatings via electron-beam evaporation

**DOI:** 10.1007/s10856-022-06679-y

**Published:** 2022-06-23

**Authors:** Xiaoyu Zhang, Yaoxu Li, Xiaobing Luo, Yumei Ding

**Affiliations:** 1 School of Energy and Power Engineering, Huazhong University of Science and Technology, 430074 Wuhan, China; 2 Department of Stomatology, Union Hospital, Tongji Medical College, Huazhong University of Science and Technology, 430022 Wuhan, China; 3 School of Stomatology, Tongji Medical College, Huazhong University of Science and Technology, 430030 Wuhan, China; 4 Hubei Province Key Laboratory of Oral and Maxillofacial Development and Regeneration, 430022 Wuhan, China

## Abstract

Antibacterial activity is one of the most vital characteristics for Titanium (Ti) dental implants. Coating antibacterial material onto Ti surfaces is an effective approach to enhance their intrinsic antibacterial ability. However, a cost-effective but efficient coating strategy for realizing this objective still remains challenging. In this study, we proposed a novel implant surface modification strategy for coating silver nanoparticles onto the porous Ti surface via a facile electron beam evaporation (EBE) approach. Porous Ti surfaces were firstly prepared by sand-blasting large grit acid-etching (SLA) process. Then, the silver nanoparticles coating thickness on the porous Ti surface was adjusted and optimized by altering the duration of EBE process. Consequently, composite porous Ti surfaces with different silver thicknesses were synthesized. Polished Ti (PT) surface without SLA or EBE process was also prepared as the controlled blank group. The surface characterizations were analyzed by SEM, AFM, and XPS. After that, the antibacterial properties of all groups were tested with bacteria counting method, bacterial viability test, live/dead bacterial staining, and SEM examination. Results show that silver nanoparticles were uniformly distributed on the porous Ti surfaces after the SLA and EBE processes. After being incorporated with silver nanoparticles, the composite surfaces successfully inhibited the growth of Escherichia coli (*E. coli*) and Staphylococcus aureus (*S. aureus*). The antibacterial ratio (AR) values of SLA-Ag groups increased with the increasing silver thickness and are significantly higher than those of PT and SLA groups. Therefore, by the SLA and EBE processes, the composite porous Ti surfaces modified with silver nanoparticles coatings demonstrate superior antibacterial property compared with pure Ti surfaces, which is highly promising for enhancing the antibacterial functions of dental implants.

**Figure:**
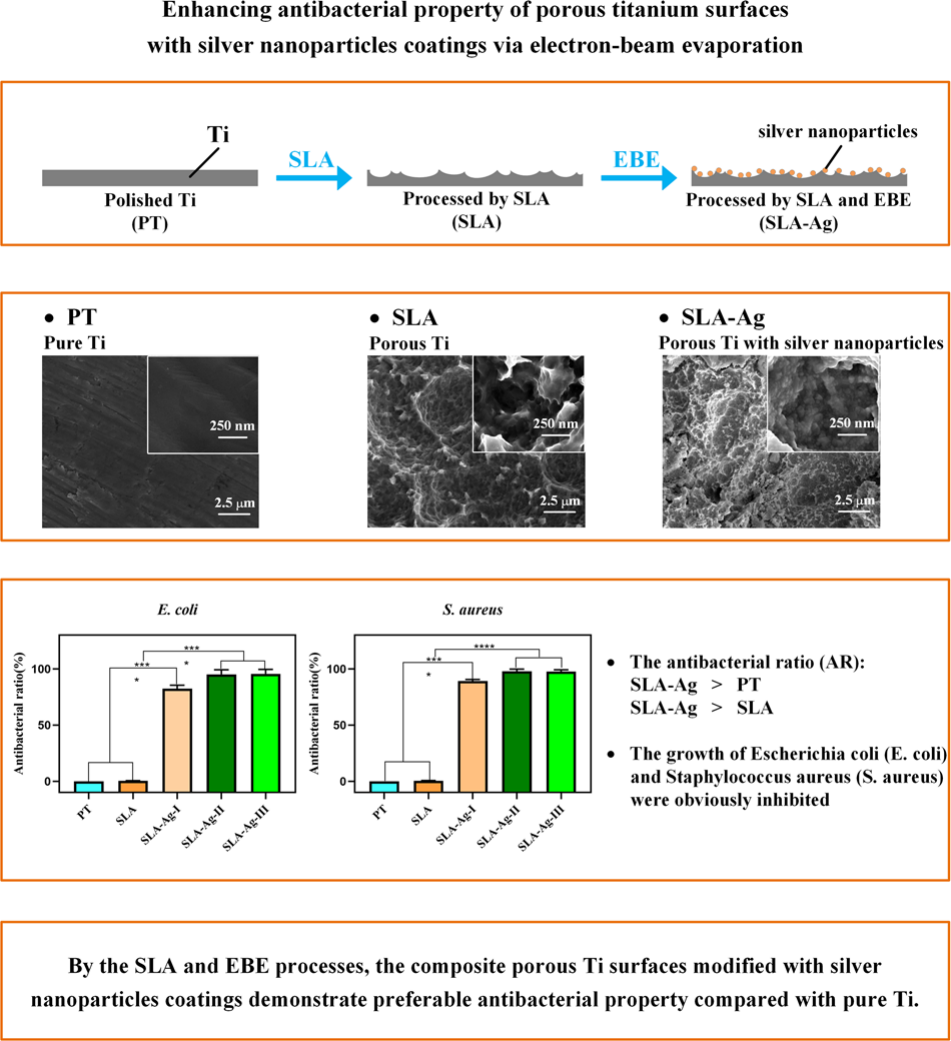
Graphical abstract

Graphical abstract

## Introduction

Titanium (Ti) has been successfully used in the field of orthopedic and dental implant due to its excellent physical and biological properties, such as high deformation and fatigue resistance, low specific gravity, and remarkable biocompatibility [1, 2]. However, the biological inertia of Ti would inhibit the osseointegration between the metal and bone tissue in some degree [3]. Therefore, many surface modifications have been proposed to improve the osseointegration between Ti and bone tissue. Sand-blasting large grit acid-etching (SLA) process is one of the most commonly used surface treatment techniques of implants [4]. This treatment can improve the surface roughness of implants and increase the contact area between Ti surface and tissue, thus promoting the osteogenic differentiation of osteoblasts and the occurrence of osseointegration [5].

Another disadvantage of Ti implants is the lack of antibacterial properties, which leads to a relatively high probability of postoperative infection [6]. Infection is the most common risk factor for dental implants that may cause peri-implant inflammation. The failures of implantation still frequently occur in clinical surgery due to the infection-related complications such as peri-implantitis. According to previous research, the incidence of peri-implantitis has reached 45%, which seriously decreases the effectiveness of implantation and increases the costs during subsequent treatment [7, 8]. Therefore, it is of clinical significance to improve the antimicrobial properties of Ti implants. Since the development of implants for decades, antibacterial experiments to prevent bacterial colonization and biofilm formation have been widely carried out. Generally, antibacterial agents are used to reduce the infection-related complications. However, the impact of antibacterial agents is poor because of the existence of bacterial biofilm [9, 10], which can protect bacteria from the action of antibacterial agents and host immune defense. As reported previously [11, 12], the concentration of antibacterial agents to inhibit the bacterial growth in the biofilm is 1000 times higher than that of planktonic bacteria without biofilm. In addition, the resistance of bacteria in the biofilm to antibacterial agents gradually increases over time. Thus, the existence of bacterial biofilm greatly decreases the effectiveness of antibacterial agents. Besides, it is difficult to detect the early invasion of bacteria, which easily results in the formation of bacterial biofilm.

Modifying Ti surface with antibacterial coatings can prevent the adhesion and proliferation of bacteria, thus inhibiting the formation of bacterial biofilm and reducing the risk of bacterial infection [13]. As a common antibacterial metal, silver has been used in surface modification to enhance the antibacterial properties of Ti implants in recent years [14]. Silver nanoparticles can eradicate a variety of bacteria and fungi even at a low concentration because of the remarkable antimicrobial activity [15]. Silver nanoparticles exhibit a larger surface area and higher antimicrobial activity than the bulk Ag [16]. In addition, silver nanoparticles have demonstrated low immunological response and cytotoxicity [17]. Therefore, silver nanoparticles coatings have been widely applied to increase antibacterial properties in extensive fields, such as drug delivery, medical imaging, surgical mesh, decoration of orthopedic and dental implants [18].

Various approaches have been investigated to modify silver nanoparticles onto Ti substrates. Specifically, in situ synthesis and physical techniques are the main strategies [19]. The former method is to immerse the Ti substrate in a silver ions (Ag + )-containing solution, and then convert the Ag+ on the substrate into silver nanoparticles using ultraviolet (UV) light or reducing agents. While, those silver nanoparticles prepared by in situ synthesis release quickly due to the weak physical adsorption to Ti substrate, resulting in the increase of free silver nanoparticles. Subsequently, Ag+ formed by the oxidation of free silver nanoparticles adhere to adenosine triphosphate (ATP) synthase on bacterial membrane followed by the instability of intracellular ATP depletion level and plasma membrane potential, which leads to the death of bacteria [20]. However, the quick release of Ag+ and excessive bactericidal effect would raise safety concerns. Therefore, to improve the biological safety and biocompatibility of silver nanoparticles modified implants, physical methods have been used to embed silver nanoparticles into implants such as ion implantation and magnetron sputtering [21–24]. Ion implantation is a common technique to implant metal materials onto the surface of substrate, but it is difficult to precisely control the uniformity of the coating. Magnetron sputtering allows the synthesis of thin films up to microscale at the laboratory level. Therefore, this technique cannot meet the requirements when preparing films that require more precision such as nanoscale. Electron beam evaporation (EBE) is a kind of physical vapor deposition that can accurately control the high-energy electrons to bombard the target material (such as silver) in the crucible by using the electromagnetic field, so that the target material is melted and deposited on the substrate (Ti). High-precision and uniform silver nanoparticles film up to nanoscale could be obtained by EBE through precisely controlling the evaporation parameters [25]. Therefore, EBE is a facile and efficient method to manufacture silver nanoparticles coatings on Ti surfaces. To the best of our knowledge, the effectiveness of silver nanoparticles coating realized by EBE approach has not been investigated in dental implant application.

Thus, we present a surface modification strategy to promote the antibacterial property of porous Ti implant with silver nanoparticles coating via the EBE approach. Firstly, porous Ti surfaces were prepared by the SLA process to simulate the surface roughness of dental implants, followed by silver nanoparticles aggregation embedded on porous structures via the EBE process. The thickness of silver nanoparticles coating was exquisitely adjusted by controlling the duration of EBE. Then the morphology, chemical and physical properties of the composite surface were evaluated. Finally, the antibacterial activities of the surfaces were investigated by inoculation with Escherichia coli (*E. coli*) and Staphylococcus aureus (*S. aureus*) bacteria.

## Materials and methods

### Materials preparation

Commercially pure TA1 Ti (purity>99.85%, Baoji Ti Industry, China) was manufactured into disks with the diameter of 15 *mm* (similar to the diameter of each hole of a 24-well plate) and thickness of 1 *mm* for use as the substrate. Three surface treatments were applied to the Ti substrates, namely machined polish (PT), sand-blasting large grit acid-etching (SLA), sand-blasting large grit acid-etching process with silver nanoparticles coating (SLA-Ag). In our study, the PT samples are used as the controlled blank group, and the experimental groups are divided into SLA (0 *nm*), SLA-Ag-I (3 *nm*), SLA-Ag-II (15 *nm*), and SLA-Ag-III (75 *nm*) according to the thickness of silver nanoparticles coating.

All the disks were mechanically polished with different SiC sandpapers (#400, #800, #1200), followed by ultrasonic cleaning with acetone, anhydrous alcohol, and deionized water in sequence for 10 min each. Finally, all the samples were air dried at room temperature, after which the preparation of PT group was finished and the SLA process was conducted.

The SLA surfaces were prepared through the large grid sand-blasting and acid-etching processes. The samples were sandblasted by aluminum oxide grit (#60) for 30 s and then acid-etched by 20% H_2_SO_4_ at 55 °C for 20 min. After ultrasonic cleaning and air drying, the preparation of SLA group was finished and the EBE process was carried out.

The SLA-Ag surfaces were prepared by large grid sand-blasting and acid-etching processes as described for SLA group and then manufactured using an EBE system (Ohmiker-50B, Cello Technology Co. Ltd). A high purity silver (99.99%) target was used to deposit silver nanoparticles coatings onto the prepared SLA surfaces. The thickness of silver was exquisitely adjusted by controlling the duration of EBE process. As a result, SLA-Ag group can be divided into SLA-Ag-I (3 *nm*), SLA-Ag-II (15 *nm*), and SLA-Ag-III (75 *nm*) according to the thickness of silver nanoparticles coatings.

### Surface characterization

The morphology of the treated surfaces in different groups were observed by scanning electron microscopy (SEM). Field-emission scanning electron microscopy (FSEM, Nova NanoSEM 450, FEI, USA) was used to characterize the morphologies of silver nanoparticles at an accelerating voltage of 10 kV. The surface roughness of the samples was characterized by atomic force microscopy (AFM, SPM-9700HT, Shimadzu, Japan). The following parameters were calculated: Sa (the arithmetic mean roughness), Sq (the root-mean-square roughness), and D_*f*_ (the fractal dimension) [26].

The chemical state and elemental depth profile were determined by X-ray photoelectron spectroscopy (XPS, AXIS-ULTRA DLD-600W, Shimadzu, Japan) to validate the existence and various amounts of silver in those different groups. The measured samples are solid disks with the diameter of 15 *mm* and thickness of 1 *mm*. A monochromatized Al Kα was used to irradiate the samples with the photon energy of 1486.6 *eV* and the X-ray spot size of 300 × 700 *μm*. Besides, the expected resolution of the measurement was 0.45 *eV*. In terms of data processing, the software of XPSPEAK41 was use to analyze the measurement results. The energy scale of the XPS spectra was corrected using the binding energy of adventitious carbon (C 1 s at 285 *eV*).

### Antibacterial test

Escherichia coli (*E. coli*) and Staphylococcus aureus (*S. aureus*) are common pathogens of medical implant infection which represent strains of gram-negative and gram-positive respectively [27]. In this study, *E. coli* (ATCC 25922) and *S. aureus* (ATCC 25923) were used to assess the antibacterial effect of various groups of the samples. *E. coli* cells were cultured in a Luria–Bertani (LB, L3522, Sigma-Aldrich, USA) broth or LB agar (A1296, sigma-Aldrich, USA) plates, and *S. aureus* cells were cultured in a Trypticase soy broth (TSB, 22092, Millipore, USA) or TSB agar plates (146317, Millipore, USA). The antibacterial activities of all the samples were tested by bacterial counting method, bacterial viability tests, live/dead staining, and SEM examination. Before inoculation with bacteria, all the samples were placed in a 24-well plate and sterilized by UV radiation for 30 min at the distance of 10 cm.

### Bacterial counting method

*E. coli* and *S. aureus* were grown overnight in LB and TSB medium and then resuspended in phosphate buffer saline to a concentration of 10^7^ CFU/mL. 1 mL of the above bacterial solution was inoculated on the surface of each sample, after which the samples were incubated at 37 °C for 24 h. After the incubation, the bacteria on the surface of each sample were collected and recultivated on agar plates respectively. After incubating at 37 °C for another 24 h, live bacteria were visualized by a gel imaging system (ChemiDoc^™^ MP System, 1708280, Bio-Rad, USA), and the live bacteria count was performed in accordance with the National Standard of China GB/T 4789.2 protocol. The antibacterial ratio (AR) is measured on the basis of the following formulas: AR = (CFU of A – CFU of B)/CFU of A × 100%, where A represents PT, and B represents one of SLA, SLA-Ag-I, SLA-Ag-II, and SLA-Ag-III respectively.

### Bacterial viability test

Above all, 1 mL bacterial solutions (concentration: 10^7^ CFU/mL) were cultured on the surfaces of all the samples in a 24-well plate at 37 °C for 24 h. Then, the alarmarBlue assay (DAL1025, Thermo Fisher Scientific, USA) was carried to measure the viabilities of each sample in the dark according to the manufacturer’s instructions. In brief, 90 *μ*L bacterial solution was firstly transferred to a 96-well plate from the 24-well plate, and then 10 *μ*L reagent was added into the 96-well plate after the reagent restored to the room temperature. After incubation at 37 °C for 4 h, the fluorescence intensity of the solution in 96-well plate was finally determined by microplate reader (Synergy H1, BioTek, USA) at the excitation wavelength of 560 *nm* and emission wavelength of 590 *nm*.

### Live/dead bacterial staining

Here, 100 *μ*L of above bacterial solution was dropped on the surfaces, and then the samples were incubated in a 24-well plate at 37 °C for 24 h. After that, the samples were stained by the Live/Dead^®^ Baclight^™^ Bacterial Viability Kits (L13152, Thermo Fisher Scientific, USA) in the dark according to the instructions of product information. Initially, one tube of component A (orange bone body) and one tube of component B (red bone body) were mixed with 5 mL sterilized distilled water. Subsequently, 500 *μ*L of the above mixture was added onto the surfaces, after which the samples were incubated in dark for 15 min at room temperature. With all the previous preparations being completed, the distribution of live/dead bacteria on each sample was observed using a fluorescence microscope (IX53, Olympus, Japan).

### SEM examination

In the SEM (SU8010, HITACHI, Japan) examination, 100 *μ*L of bacterial solution at a concentration of 1 × 10^7^ CFU/mL was introduced onto various samples and incubated at 37 °C. 24 h later, the samples were fixed with 2.5% glutaraldehyde followed by dehydrated in a series of ethanol solutions [28]. Finally, the morphology and growth status of bacteria on the surface of Ti samples were observed by SEM.

### Statistical analysis

Each sample ran the experiments for three times independently. The data were expressed as the mean ± standard deviation (SD). A one-way analysis of variance (ANOVA) following a Student–Newman–Keuls post-hoc test was used to determine statistical significance. A difference with *P* < 0.01 was considered as significant and indicated by “**”. In addition, *P* < 0.0001 was indicated by “****”.

## Results and discussion

### Surface characterization of different Ti surfaces

**Fig. 1:**
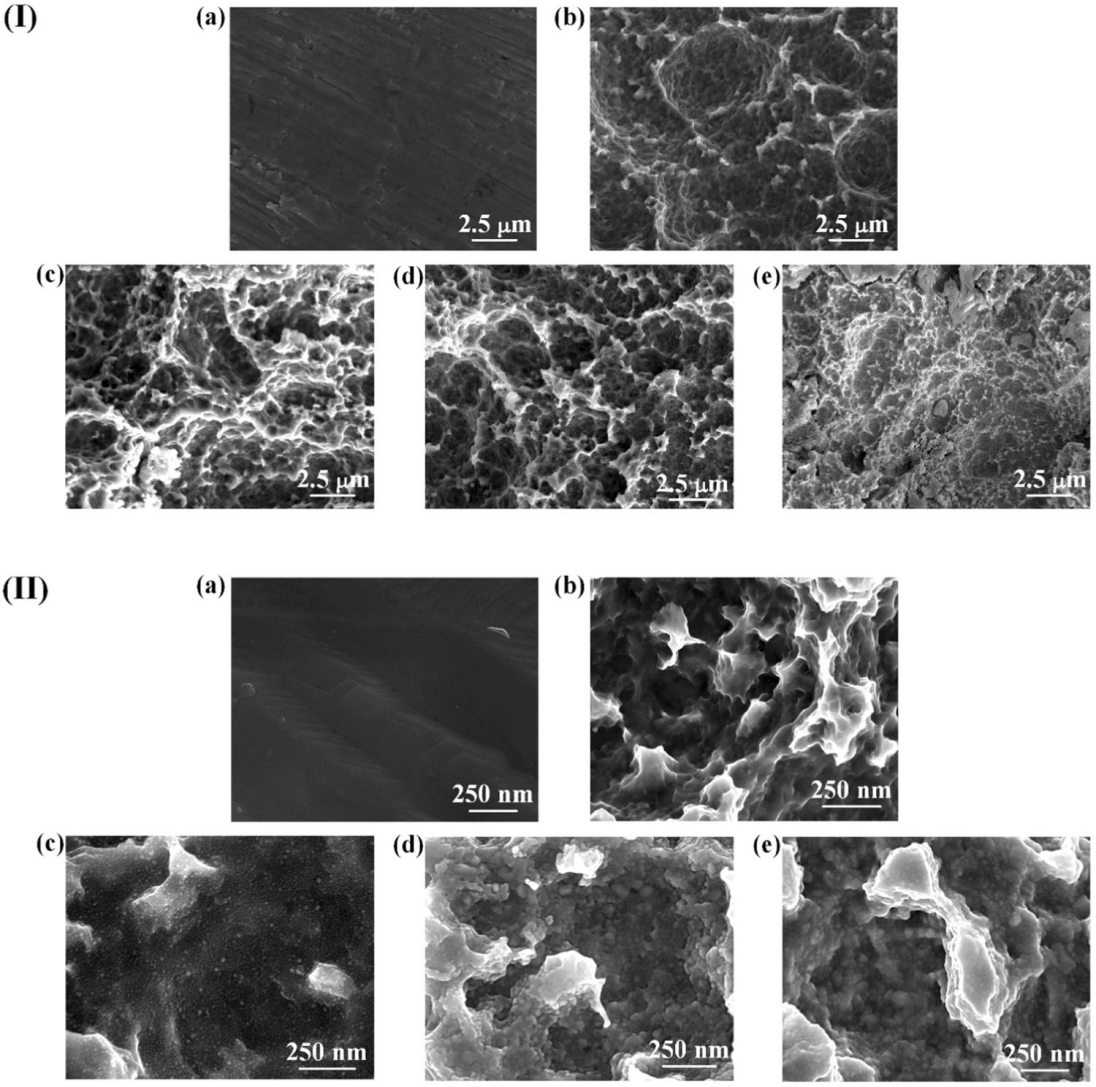
SEM images with the magnifications of (I) ×10,000 and (II) ×80,000 for the samples of (**a**) PT, (**b**) SLA, (**c**) SLA-Ag-I, (**d**) SLA-Ag-II, and (**e**) SLA-Ag-III. Pictures with a low magnification (×10,000) show the overall microscale topography, while those with a high magnification (×80,000) demonstrate the existence of nanoparticles

The topography of different Ti surfaces is shown in Fig. 1(I). Under a low magnification of ×10,000, the surface of control group (PT) appears to be extremely flat, while the surfaces of four experimental groups (SLA, SLA-Ag-I, SLA-Ag-II, and SLA-Ag-III) show similar microporous structure. Thus, to promote the authenticity and universality of our experiment, SLA process is a necessary step to prepare microporous surface morphology similar to clinical implants. The microporous structure consists of numerous micron-sized holes with the average diameter around 0–3 *μ*m. under a higher magnification of ×80,000, there is no adhesion of particles on the surfaces of PT and SLA. However, as shown in Fig. 1(II)(c)(d)(e), the surfaces of three SLA-Ag groups (SLA-Ag-I, SLA-Ag-II, and SLA-Ag-III) are uniformly coated with nanoscale granular aggregation (subsequently determined to be silver nanoparticles via XPS), which indicates the success coating of silver nanoparticles. The average diameters of nanoparticles in SLA-Ag-II and SLA-Ag-III are obviously larger than that in SLA-Ag-I, while the diameter in SLA-Ag-III is slightly larger than that in SLA-Ag-II. Thus, the more silver nanoparticles were plated, the denser silver nanoparticles coating was prepared. The average diameter of silver nanoparticles increases with the increasing silver thickness, eventually reaching a maximum value. In addition, Fig. 1(II)(c)(d)(e) show that the existence of silver nanoparticles didn’t eliminate the microporous structure. Therefore, the addition of silver nanoparticles will not greatly affect the roughness of the surface.

Roughness is regarded as a quantification of surface topography. Table 1 displays the Sa, Sq, and D_*f*_ values obtained from AFM. The Sa of group PT, SLA, SLA-Ag-I, SLA-Ag-II, and SLA-Ag-III are 23.048, 254.877, 263.226, 333.289, and 376.391 *nm* respectively, while the Sq are 28.540, 329.652, 330.314, 406.871, and 500.828 *nm*. It shows that the surface roughness of experimental groups (SLA, SLA-Ag-I, SLA-Ag-II, and SLA-Ag-III) are significantly higher than that of control group (PT). Moreover, the surface roughness of experimental groups demonstrates a gradually increasing trend with the increasing silver thickness (SLA < SLA-Ag-I < SLA-Ag-II < SLA-Ag-III). Here the fractal analysis was also conducted. In this method, the size of cube cells and the number of cells needed for coating are determined first. Then, data points of the cell size and number of cells calculated are aligned to a double-logarithmic fit. The negative slope is used as the fractal dimension (D_*f*_). In this algorithm, D_*f*_ varies between 2 (flattest) and 3 (roughest). The D_*f*_ of group PT, SLA, SLA-Ag-I, SLA-Ag-II, and SLA-Ag-III are 2.002, 2.116, 2.174, 2.194, and 2.203 respectively. The results show the same tendency as those of Sa and Sq. Consistent with the results in SEM, the AFM results quantitatively confirm that the presence of silver nanoparticles didn’t eliminate the microporous structure but further increased the surface roughness. Therefore, the SLA process significantly increased the surface roughness, and the silver nanoparticles coatings added by the EBE process further contributed to the increase of surface roughness. As it is known, rough surface exhibits larger surface area to promote osteogenesis and bone-implant integration, while osteoclastic activity and formation are suppressed [29–31]. Therefore, in our experiments, the SLA process is an essential step to significantly increase surface roughness of Ti substrates. In addition, within the scope of this experiment (the thickness of silver nanoparticles coating ≤75 *nm*), the silver nanoparticles coatings realized by the EBE process also show a positive effect on improving the roughness.

**Table 1:** The Sa and Sq values of different Ti surfaces

Group	PT	SLA	SLA-Ag-I	SLA-Ag-II	SLA-Ag- III
Roughness					
Sa [*nm*]	23.048	254.877	263.226	333.289	376.391
Sq [*nm*]	28.540	329.652	330.314	406.871	500.828
D_*f*_	2.002	2.116	2.174	2.194	2.203

**Fig. 2:**
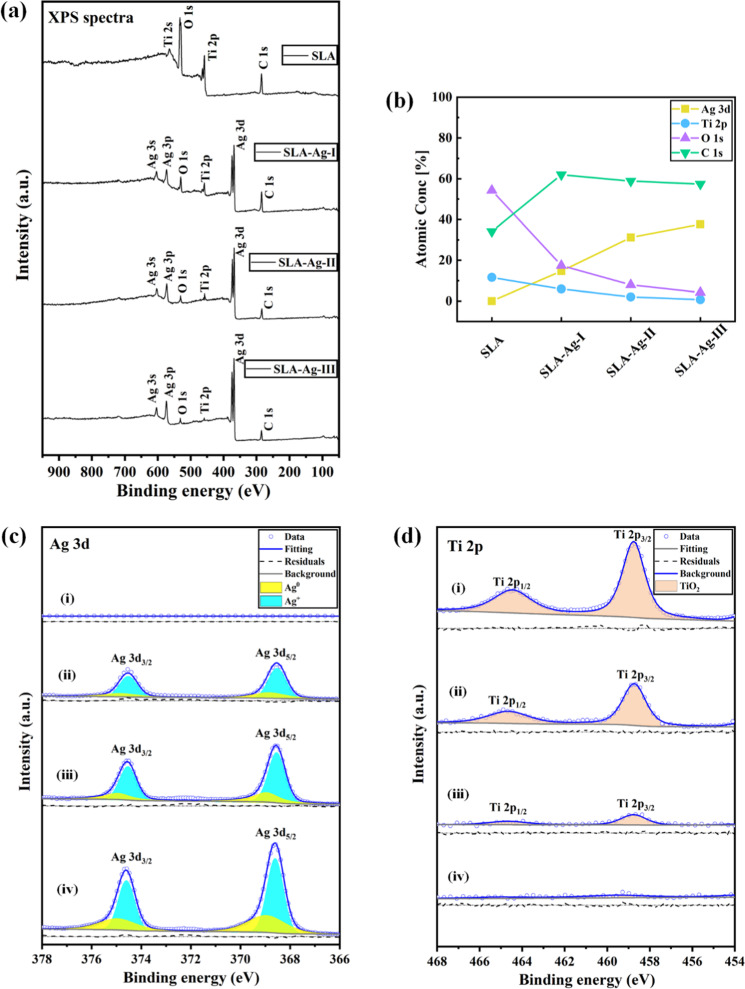
XPS results of four experimental groups (SLA, SLA-Ag-I, SLA-Ag-II, and SLA-Ag-III). **a** Wide scan XPS spectra. **b** XPS depth-profile of four groups. Detailed spectra of (**c**) Ag 3d and (**d**) Ti 2p detected on (i) SLA, (ii) SLA-Ag-I, (iii) SLA-Ag-II, and (iv) SLA-Ag-III surfaces

To reveal the differences of chemical characteristics in the experimental groups, the chemical elements on the sample surfaces were further investigated by XPS. Figure 2(a) displays wide scan XPS spectra, which presents surface compositions of four experimental groups (SLA, SLA-Ag-I, SLA-Ag-II, and SLA-Ag-III). It is seen that the main components are Ti 2p, O 1 s, and C 1 s peaks in SLA group. The peak of Ti 2p exists due to the Ti substrate, while the peaks of O 1 s and C 1 s result from the atmospheric environments reacted with Ti substrate. However, Ti 2p, O 1 s, and C 1 s peaks gradually decrease in groups of SLA-Ag-I, SLA-Ag-II, and SLA-Ag-III. In addition, a significant Ag 3d peak exists in those three groups because of the silver nanoparticles coatings modified by the EBE process. Therefore, the chemical elements exist on the substrates are reasonable and agree with the actual sample preparation process. Besides, the results show that the samples are not contaminated by other irrelevant elements, which ensures the credibility of the subsequent biological experiments.

Figure 2(b) shows XPS depth-profile of four groups. The composition of the elements demonstrates that the content of silver gradually increases, and the contents of Ti and O gradually decrease, and the tendency shows the same as that in Fig. 2(a).

Wide scan XPS spectra in Fig. 2(a) demonstrate the elements composition of samples from four experimental groups. However, the valence states of elements cannot be determined. To further investigate and recognize the valence states of the crucial elements, XPS peak fitting was conducted according to relevant guidelines and advice [32, 34], and corresponding high-resolution XPS spectra of Ag 3d and Ti 2p are analyzed in Fig. 2(c) and Fig. 2(d). The binding energy difference between Ag 3d_5/2_ and Ag 3d_3/2_ is 6.0 eV, and the binding energy difference between Ti 2p_3/2_ and Ti 2p_1/2_ is 5.7 eV. As shown in Fig. 2(c)(i), the samples of SLA group don’t contain silver, while other three groups (SLA-Ag-I, SLA-Ag-II, and SLA-Ag-III) contain silver with the valence state of Ag^0^ and Ag^+^. Ag^0^ can exist on the surface due to the chemical inertness of Ag to O. Thus, Ag element exists on the surface with the chemical compositions of silver and silver oxide (AgO). Figure 2(d) shows that the samples of SLA, SLA-Ag-I, and SLA-Ag-II groups contain Ti with the chemical state of TiO_2_. Ti^0^ cannot exist on the surface because Ti is more active compared with Ag. Therefore, Ti element exists on the surface with the chemical compositions of titanium dioxide (TiO_2_). The increase of Ag content in three SLA-Ag groups demonstrates the increase of silver nanoparticles on Ti surface, which may cause different antibacterial property. However, the increase of silver nanoparticles leads to a denser coating, which reduces the exposure of Ti substrate to a certain extent. Therefore, the content of Ti in four experimental groups gradually decreases as shown in Fig. 2(d). In addition, the increase of silver nanoparticles reduces the reaction between Ti substrate and oxygen in the air due to the chemical inertness of Ag to O. Thus, the O content on the surfaces in four experimental groups also decreases in turn. The results show that the existence of silver nanoparticles may inhibit the oxidation of Ti substrate.

### Antibacterial tests of various Ti surfaces

**Fig. 3:**
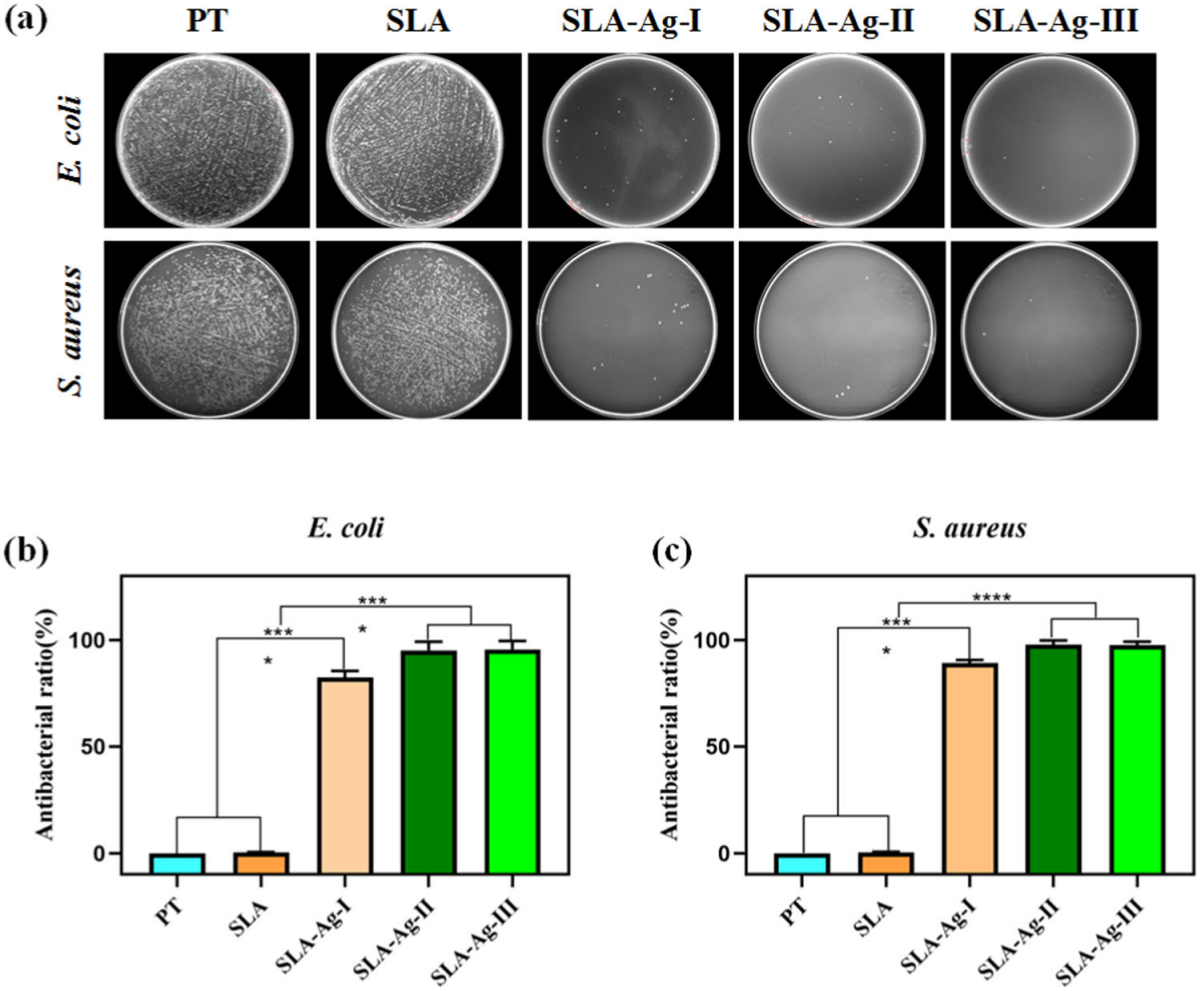
**a** Photographs of re-cultivated *E. coli* and *S. aureus* collected from the samples. Antibacterial ratio against *E. coli* (**b**) and *S. aureus* (**c**)

After 24 h of incubation on different Ti surfaces, bacteria were collected and recultivated on the agar plates overnight, after which the live bacteria were visualized by a gel imaging system. In Fig. 3(a), plenty of bacterial colonies grow on the agar plates cultured with E. coli or S. aureus dissociated from PT and SLA samples, while nearly no bacterial colonies appear on the samples of SLA-Ag-I, SLA-Ag-II, and SLA-Ag-III. The results indicate that SLA-Ag-I, SLA-Ag-II, and SLA-Ag-III show a strong bactericidal effect and nearly no live bacteria exist on their surfaces. However, the antibacterial properties of PT and SLA groups are much weaker. Bacterial counting tests further confirm the above results. Evaluated by a spread plate method, the AR is shown in Fig. 3(b) and Fig. 3(c). The ARs of *E. coli* on the samples of SLA, SLA-Ag-I, SLA-Ag-II, and SLA-Ag-III are 0.37 ± 0.17, 82.48 ± 2.50, 95.54 ± 2.11 and 96.34 ± 2.34%, respectively; and those of *S. aureus* on the samples of SLA, SLA-Ag-I, SLA-Ag-II, and SLA-Ag-III are 0.50 ± 0.16, 89.25 ± 1.15, 97.86 ± 1.65, 97.61 ± 1.37%, respectively. For both *E. coli* and *S. aureus*, the ARs of SLA-Ag groups (SLA-Ag-I, SLA-Ag-II, and SLA-Ag-III) are significantly higher than those of PT and SLA samples (*P* < 0.0001). Besides, the AR of SLA-Ag-I is substantially lower than those of SLA-Ag-II and SLA-Ag-III (*P* < 0.0001). However, there are no apparent differences on AR between either PT and SLA, or SLA-Ag-II and SLA-Ag-III.

**Fig. 4:**
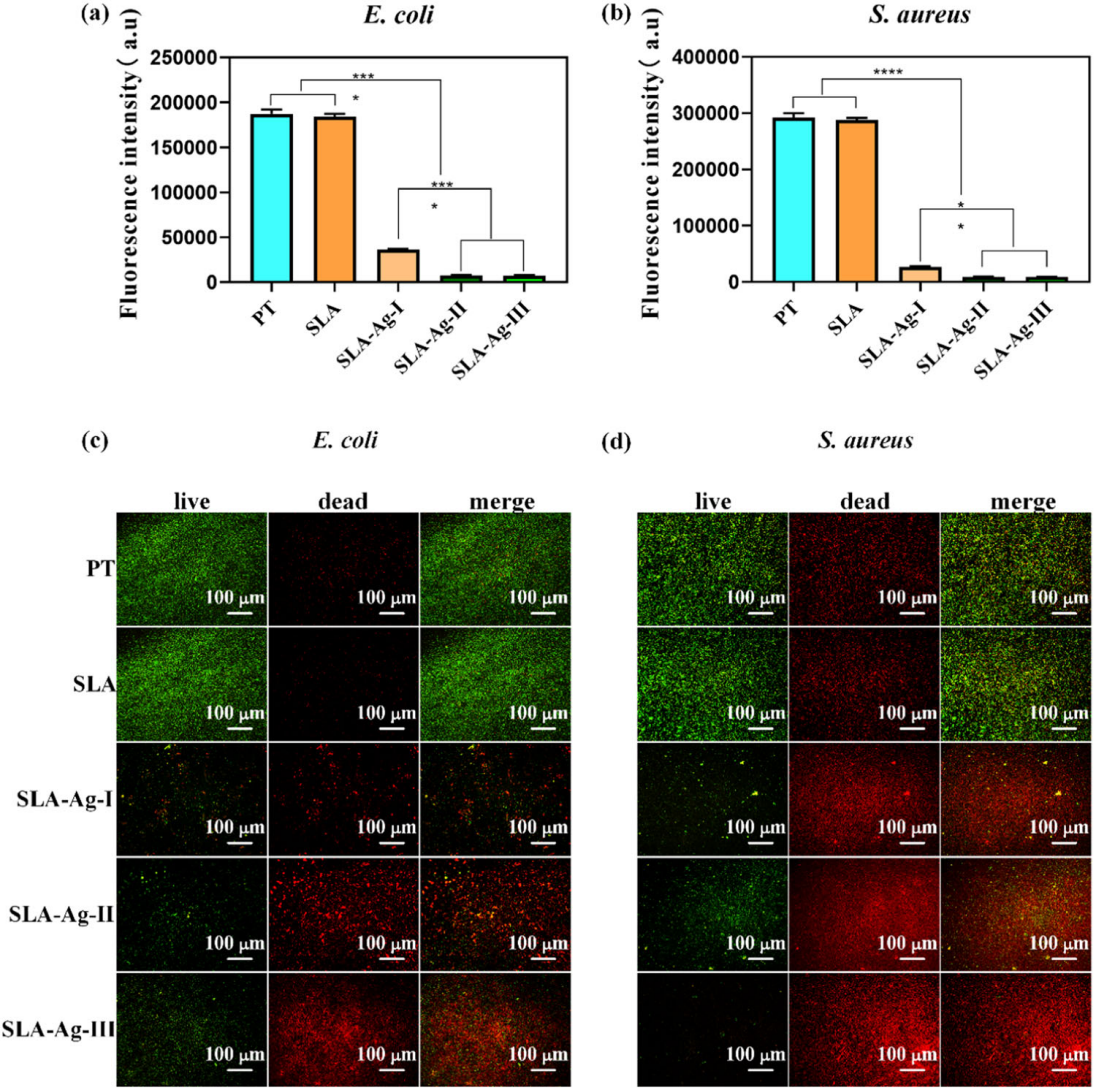
Viabilities of *E. coli* (**a**) and *S. aureus* (**b**) cultured on the samples. Fluorescence images of the live/dead-stained *E. coli* (**c**) and *S. aureus* (**d**) cultured on the samples

The viability of *E. coli* and *S. aureus* on the samples were characterized by alamarBlue assay. After the bacteria were cultured on the surfaces for 24 h, the fluorescence intensity of bacterial solution was measured. As shown in Fig. 4(a), the fluorescence intensities of PT and SLA in the *E. coli* groups are ~180,000, which are significantly higher than that of SLA-Ag-I (about 40,000) (*P* < 0.0001). In addition, the fluorescence intensity of SLA-Ag-I is much higher than those of SLA-Ag-II and SLA-Ag-III (both <10,000) (*P* < 0.0001). The results of inoculating *S. aureus* are exhibited in Fig. 4(b). It illustrates that the fluorescence intensity of PT and SLA are both around 290,000, substantially higher than that of SLA-Ag-I, which is near 26,000 (*P* < 0.0001). In addition, the fluorescence intensities of SLA-Ag-II and SLA-Ag-III are below 10,000, which are much lower than that of SLA-Ag-I (*P* < 0.01). However, for both *E. coli* and *S. aureus*, there are no apparent differences on fluorescence intensity between either PT and SLA, or SLA-Ag-II and SLA-Ag-III. As reported, the fluorescence intensity is proportional to the number of living bacteria [34]. Therefore, consistent with the results of the bacterial counting method, the SLA sample has an inapparent impact on *E. coli* or *S. aureus* compared to the PT samples. However, the samples of SLA-Ag-I, SLA-Ag-II, and SLA-Ag-III significantly inhibited the growth of bacteria. Moreover, the inhibition of SLA-Ag-II and SLA-Ag-III, on which both *E. coli* and *S. aureus* exhibited viability close to zero, was substantially stronger than that of SLA-Ag-I. Images of the live/dead-stained bacteria at a magnification of ×100 are shown in Fig. 4(c) and Fig. 4(d). For both *E. coli* and *S. aureus*, most of the bacteria survive (stained to green) on the samples of PT and SLA. However, almost all the bacteria die (stained to red) on the samples of SLA-Ag-I, SLA-Ag-II, and SLA-Ag-III. Hence, SLA-Ag groups have an obvious antibacterial effect on bacteria, while Pt and SLA almost appeared no impact.

**Fig. 5:**
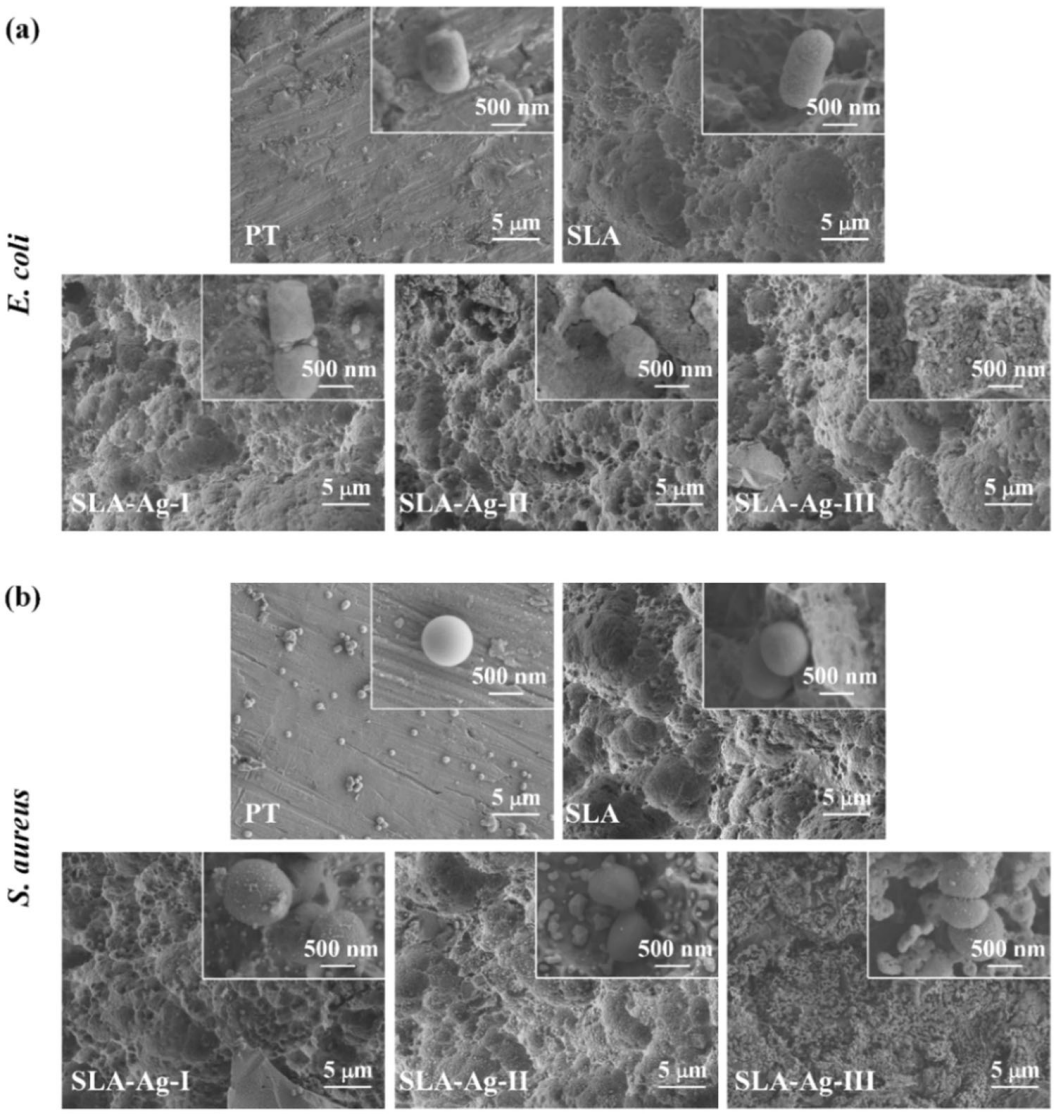
SEM images showing the *E. coli* (**a**) and *S. aureus* (**b**) cultures on various samples

The SEM images of *E. coli* and *S. aureus* on all groups are shown in Fig. 5. Many bacteria are observed on the relatively flat surfaces of PT samples, whereas there are only a few bacteria on the experimental groups (SLA, SLA-Ag-I, SLA-Ag-II, and SLA-Ag-III). Most *E. coli* and *S. aureus* cells on the PT and SLA surfaces appear normal in shape (rod-shaped for *E. coli* and spherical for *S. aureus*). In contrast, bacteria on the surfaces of SLA-Ag groups exhibit lysis and cytoplasmic leakage, indicating an obvious antibacterial effect of SLA-Ag-I, SLA-Ag-II, and SLA-Ag-III on bacteria. Although the number of bacteria on the surfaces of all groups is small, which may be related to the process of dehydration and fixation before electron microscopy, the antibacterial effect of SLA-Ag groups is obvious compared to those of PT and SLA groups.

SLA is one of the most commonly used surface treatment techniques of implants. This treatment can improve the surface roughness of implants. However, many studies declare that rough surface is conducive to the formation of bacterial biofilm [35, 36], leading to the reduction of antibacterial property. In this study, the antibacterial property of silver nanoparticles coatings is far greater than the promotion effect of roughness on biofilm formation. The surface roughness of the SLA-Ag groups increases, but the antibacterial performance improves at the same time. Hence, the preparing methods presented in this study would effectively promote the antibacterial property of implant and reduce the incidence of infection [37]. However, there are no apparent differences in antibacterial property between SLA-Ag-II group (the thickness of silver nanoparticles coating: 15 *nm*) and SLA-Ag-III group (the thickness of silver nanoparticles coating: 75 *nm*). Thus, the optimal amount of silver that is conducive to antibacterial and osteogenesis needs further exploration.

## Conclusions

In this study, a surface modification strategy is presented to promote the antibacterial property of Ti implant by fabricating composite porous surfaces with silver nanoparticles coating via SLA and EBE processes. Firstly, microporous structures close to the surfaces of clinical Ti implants were prepared through the SLA process. Then the silver nanoparticles coatings on microporous surfaces were fabricated by the EBE process to improve the antibacterial properties of porous Ti surface. The amount of silver was exquisitely adjusted by controlling the duration of EBE process. The ARs of *E. coli* and *S. aureus* on the samples of SLA-Ag groups reach 96.34 ± 2.34 and 97.61 ± 1.37%, which are significantly higher than those groups without silver nanoparticles coatings (PT and SLA). Therefore, the composite porous surfaces modified with silver nanoparticles coatings demonstrate preferable antibacterial property compared with pure Ti surfaces. The effectiveness of silver nanoparticles coating realized by EBE approach is confirmed to be valid and obvious, and thus suggests a reliable surface modification strategy to promote the antibacterial property of Ti for dental implant clinical application.
